# Mutations in m^6^A consensus motifs are suppressed in the m^6^A modified genes in human cancer cells

**DOI:** 10.1371/journal.pone.0236882

**Published:** 2020-08-13

**Authors:** Mingzi An, Huiyun Wang, Yingqian Zhu

**Affiliations:** 1 Department of Gynaecology, Chengyang People's Hospital, Qingdao, Shandong, China; 2 Department of Oncology, The Affiliated Hospital of Qingdao University, Qingdao, Shandong, China; University of Toronto, CANADA

## Abstract

*N*^6^-methyladenosine (m^6^A) is the most prevalent type of RNA modification. METTL3 in the methyltransferase complex is the core enzyme responsible for methylation. METTL3 selectively catalyzes the adenosines centered in the RRAC motif. Functional studies established that m^6^A could enhance the translation efficiency (TE) of modified genes by recruiting reader protein YTHDF1 and other initiation factors. We downloaded the m^6^A peaks in HeLa cells from a previous study and defined the m^6^A modified genes and sites. Ancestral mutations in the genic region fixed in the HeLa cell samples were defined using their mRNA-Seq data and the alignment between human and mouse genomes. Furthermore, in the small interfering (si)-*METTL3* sample, the calculated TE foldchange of all genes was compared to that in the negative control. The TE of m^6^A genes was globally down-regulated in si-*METTL3* versus control compared to the non-m^6^A genes. In m^6^A modified genes, RRAC motif mutations were suppressed compared to mutations in non-motif regions or non-m^6^A genes. Among the m^6^A genes, a fraction RRAC motif mutations negatively correlated with the TE foldchange (si-*METTL3* versus control). The TE of m^6^A modified genes was enhanced in HeLa cells. RRAC motif mutations could potentially prevent methylation of adenosines and consequently abolish the enhanced translation. Such mutations in the RRAC motif might be deleterious. Accordingly, we observed lower fractions of mutations in RRAC motifs than in other regions. This prevention of mutations in the RRAC motif could be a strategy adopted by cancer cells to maintain the elevated translation of particular genes.

## Introduction

*N*^6^-methyladenosine (m^6^A) is one of the most prevalent types of RNA modifications in animals [1–10]. METTL3 in the multi-component methyltransferase complex is the core enzyme responsible for methylation. METTL3 preferentially catalyzes the adenosine sites located in the RRAC motif (R = A or G, A = methylated adenosine). Modification of m^6^A can increase the translation efficiency (TE) of host genes when it is recognized by the reader protein YTHDF1 and the translation initiation factors are recruited [6]. This is not the only type of RNA modification that can modulate mRNA translation [11]. This phenomenon suggests a potential selection pattern for these various RNA modification events.

If the increased translation of m^6^A modified genes is beneficial, any changes that abolish this elevation would be slightly deleterious. Since the RRAC motif is necessary for methylation, we wondered whether the mutations in RRAC motifs would be deleterious and if they can be purged by natural selection. Furthermore, if a gene depends more on m^6^A modification to enhance its translation, this gene would have lower tolerance for mutations that abolish this enhancement.

A study reported that m^6^A sites in humans are generally non-adaptive and likely to be nonfunctional [12] as methylated adenosines are less conserved than unmethylated adenosines. The same group has reported similar results for other RNA modification types in humans [13]. However, for several reasons, this does not exclude the necessity to study the human m^6^A system. First, even if a type of modification is non-adaptive in humans [12, 13], the same modification could be adaptive in other species [14]. Furthermore, a small portion of the conserved m^6^A sites might be functional, even if the global methylome is nonfunctional. Additionally, given the present nonfunctional properties of human m^6^A sites, knowledge of its evolutionary traces are required to decipher how it became nonfunctional during evolution.

To test our assumption that the mutations that abolish the m^6^A RRAC motif are be deleterious, we downloaded the m^6^A peaks in HeLa cells generated in a previous study [6] and defined the m^6^A modified genes and sites. We next called variants/mutations in the genic region from the HeLa cell samples by using their mRNA-Seq data (since we do not need data from the intergenic region, DNA-Seq is not necessary for variant calling here). Whole human and mouse genome alignment (genomes were downloaded from the UCSC genome browser) was used to infer the ancestral state of the mutation. Furthermore, we calculated the TE foldchange of all genes in small interfering (si)-*METTL3* versus in the control samples. The results revealed that the translation of m^6^A modified genes is down-regulated in si-*METTL3* compared with that in the unmodified non-m^6^A genes, which agrees with the established theory [6].

We then focused on mutations that could potentially abolish the RRAC motifs. There is a lower fraction of mutations in the RRAC motif in m^6^A genes compared to in the non-motif regions and/or in-m^6^A genes. Furthermore, we tested the correlation between the fraction of mutations in the RRAC motif and the TE foldchange of m^6^A genes in si-*METTL3* versus in control. The negative correlation between these two variables indicated that the genes highly regulated by m^6^A have low tolerance for mutations in the RRAC motifs.

We propose that in HeLa cells, mutations in the RRAC motif would potentially prevent the methylation of adenosines and consequently abolish the enhancement of mRNA translation. These deleterious mutations in RRAC motifs are suppressed, especially in genes that are highly regulated by m^6^A. The suppression of mutations in the RRAC motif might be a strategy adopted by cancer cells to maintain the elevated translation of particular genes. The findings of this study broaden the understanding of the dynamics of mutations affecting the m^6^A motif and are therefore valuable to the field of cancer biology and the m^6^A community.

## Materials and methods

### Next Generation Sequencing (NGS) data

NGS data form normal HeLa cells or HeLa cells with si-*METTL3* were downloaded from a previous study [6] under accession number GSE63591. The NGS data contained mRNA-Seq and Ribo-Seq (ribosome profiling followed by deep sequencing) information [15] which enabled us to define the TE of each gene. The adenosine sites in the RRAC motif (R = A or G, A = methylated A; if located in m^6^A peaks) were systematically recognized as m^6^A modification sites. The genomic coordinates of the m^6^A peaks are provided as supplementary files in the original study [6].

### Assigning m^6^A sites to human genes

We downloaded the hg19 human genome sequence (fasta format) and the gene annotation files (gtf format) from the UCSC genome browser [16]. The m^6^A sites were annotated according to their genomic coordinate information and only the m^6^A sites in the genic regions were considered. Each m^6^A site was assigned a gene ID and a gene name. The few human genes that overlapped with each other were not considered.

Codes:

bedtools intersect -a m6A.peak -b hg19.gtf–wa–wb > m6A.peak.annotation

### NGS data processing

The sequencing reads were aligned to the human genome (hg19) using STAR (version 2.3) [17]. Default parameters were used. The read count for each gene (including any overlap with all exons) was determined using htseq-count version 0.9 [18] with default parameters.

Codes:

STAR—runMode genomeGenerate—genomeFastaFiles hg19.fasta—sjdbGTFfile hg19.gtf—runThreadN 2—sjdbOverhang 50

STAR—runThreadN 2—genomeDir./—readFilesIn sampleX.fq—outFileNamePrefix sampleX

htseq-count -t exon sampleX.sam hg19.gft > sampleX.count

### TE of genes

We utilized xtail [19] to determine the TE of each gene or the TE foldchange between si-*METTL3* and the control libraries. When calculating TE, only the reads mapped to coding sequence (CDS) were counted.

### Variant calling

Variants from the mRNA-Seq data were called using samtools version 1.4 [20] using a minimum coverage of 10. Variants with alternative allele frequencies (<0.9) were discarded. The remaining variants were regarded as fixed mutations in the HeLa cell sample studied.

Codes:

samtools mpileup sampleX.sam > sampleX.vcf

awk -F “,” ‘$2> = 10 && $3/$2> = 0.9’ sampleX.vcf > sampleX.variants

### Inference of ancestral state of the mutations

**Fig 1:**
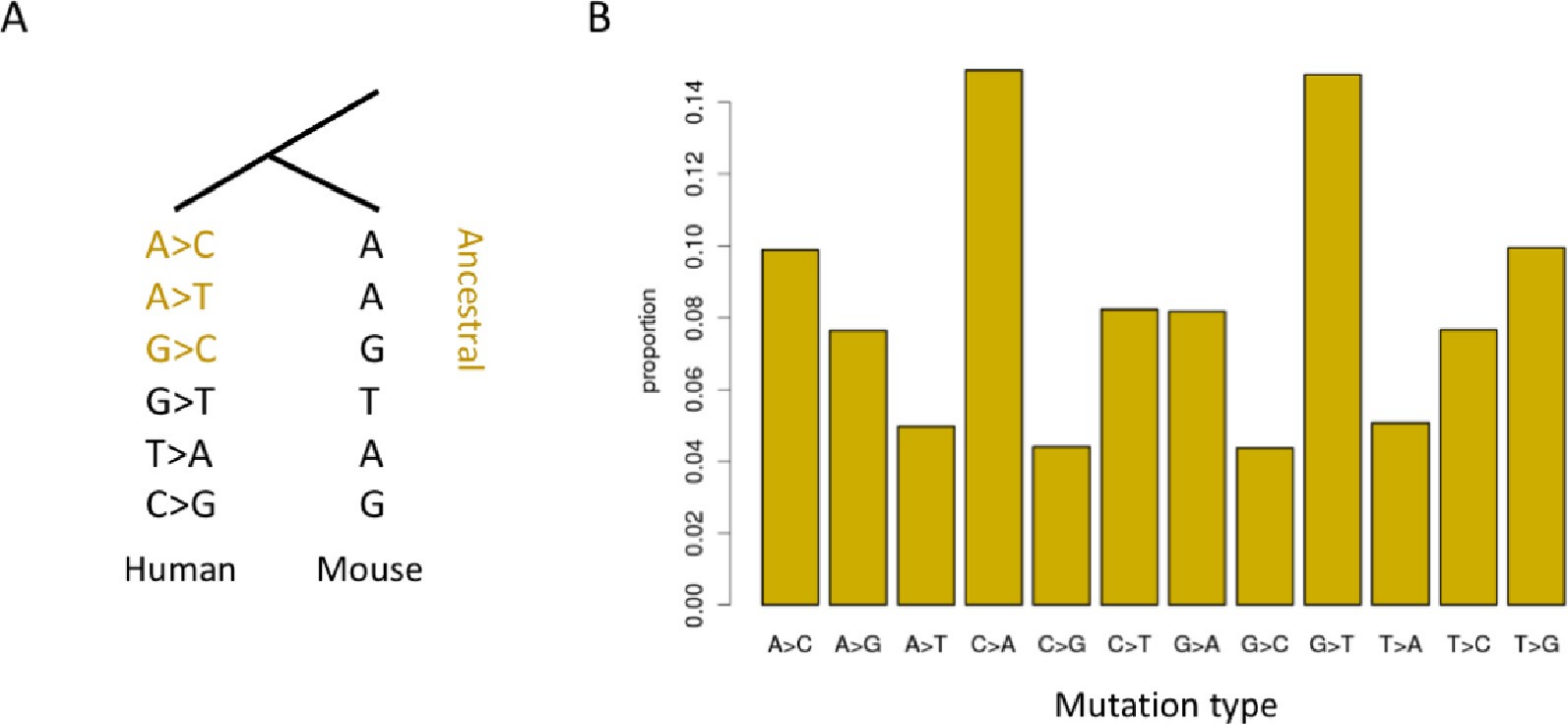
The mutation profile in HeLa cells. (A) Inferring the ancestral state of mutations; (B) Proportion of different types of mutations.

We used the liftover tool (from hg19 version to mm10 version) downloaded from the UCSC genome browser [16] to transfer the genomic coordinates from human to mouse. For a given human mutation site, if the base in mouse was identical to the reference sequence in the human genome, this mutation was defined as ancestral (Fig 1A).

Codes:

LiftOver hg19.coordinates hg19tomm10.chain mm10.coordinates

Bedtools getfasta -fi mm10.fasta -bed mm10.coordinates -fo mm10.coordinates.base

### Control of mutation bias in RRAC motifs

When mutations in the RRAC motif (R = A or G) were assessed, only those mutations that abolished this motif were counted. For example, AGAC and AAAC both conform to the RRAC motif, so the G>A or A>G mutations at the first two positions do not destroy this motif and were, therefore, not counted. To conduct an unbiased analysis across all regions and genes, the G>A or A>G mutations (single nucleotide polymorphisms, SNPs) were discarded in all analyses related to fractions. Thus, only the other mutation types were included in the calculation of the fraction. Another potential bias stipulated that the methylated RRAC motifs not have mutations on the adenosine sites (otherwise they could not be methylated). To conduct an unbiased comparison, we chose only those unmethylated RRAC motifs that lacked variants on adenosine sites.

### Statistical analyses

R language version 3.3.3 was used for statistical analyses. Some of the figures were also plotted in the R environment. The bash codes, which are the minimal datasets underlying this study, are provided after each subsection of Materials and Methods.

## Results

### Mutations in HeLa cells and RRAC motifs

We retrieved NGS data from normal HeLa cells [6] and conducted variant calling in the genic regions. In total, 729,781 reliable ancestral mutations were obtained from the mRNA-Seq data (Fig 1A). Their mutation types were profiled (Fig 1B). C>A and G>T mutations were found to be the most frequent (Fig 1B).

**Fig 2:**
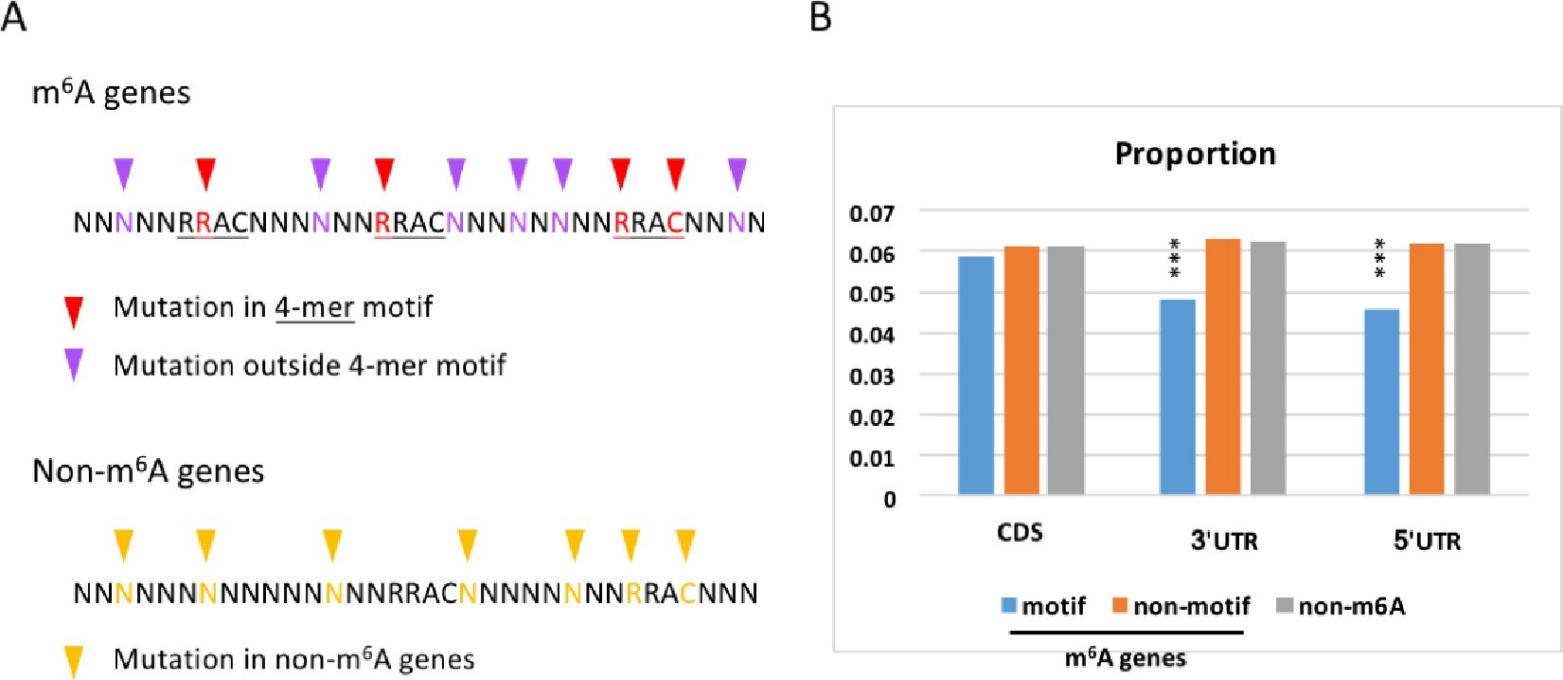
Fraction of mutations in different categories. (A) Diagram showing how we define mutations in RRAC motifs and other regions; (B) Fraction of mutations in m^6^A genes and non-m^6^A genes, in RRAC motifs and outside RRAC motifs, and in the RRAC motifs in different functional categories (CDS, 5’UTR and 3’UTR). “***” means p-value < 0.001 under Chi-square test. The “motif” group is compared with the “non-motif” and “non-m^6^A” group.

We next defined m^6^A and non-m^6^A genes according to whether the m^6^A peaks appear in a gene. The 6,252 m^6^A genes contained approximately 18,000 m^6^A sites located in the RRAC motifs. For m^6^A genes, we calculated the fraction of SNPs in the RRAC motif and outside the motif (Fig 2A). The fraction of SNPs in a region equaled the number of mutations in the region/length of the region. For example, if there were one mutation in an RRAC motif (C>G mutation on C), then the fraction would be 1/4. Only those mutations that abolished this motif were counted (A>G and G>A were excluded, as detailed in the Materials and Methods). Only unmethylated RRAC motifs without mutations on the adenosine sites were chosen (as also detailed in the Materials and Methods). Following this, the same fraction was calculated for non-m^6^A genes (Fig 2A).

Interestingly, the fraction of mutations was significantly lower in the RRAC motifs in the 3′ untranslated region (UTR) and 5′UTRs of m^6^A genes compared to the fraction in the non-motif regions or non-m^6^A genes (Fig 2B). It has been established that m^6^A modifications in both 5′UTRs [9, 21] and 3′UTRs [6] are important for translation regulation. Thus, we surmised that the presently observed difference in mutation fractions might be related to the translation regulation conferred by m^6^A.

### Changes in TE are correlated with the fraction of mutations in RRAC motifs

**Fig 3:**
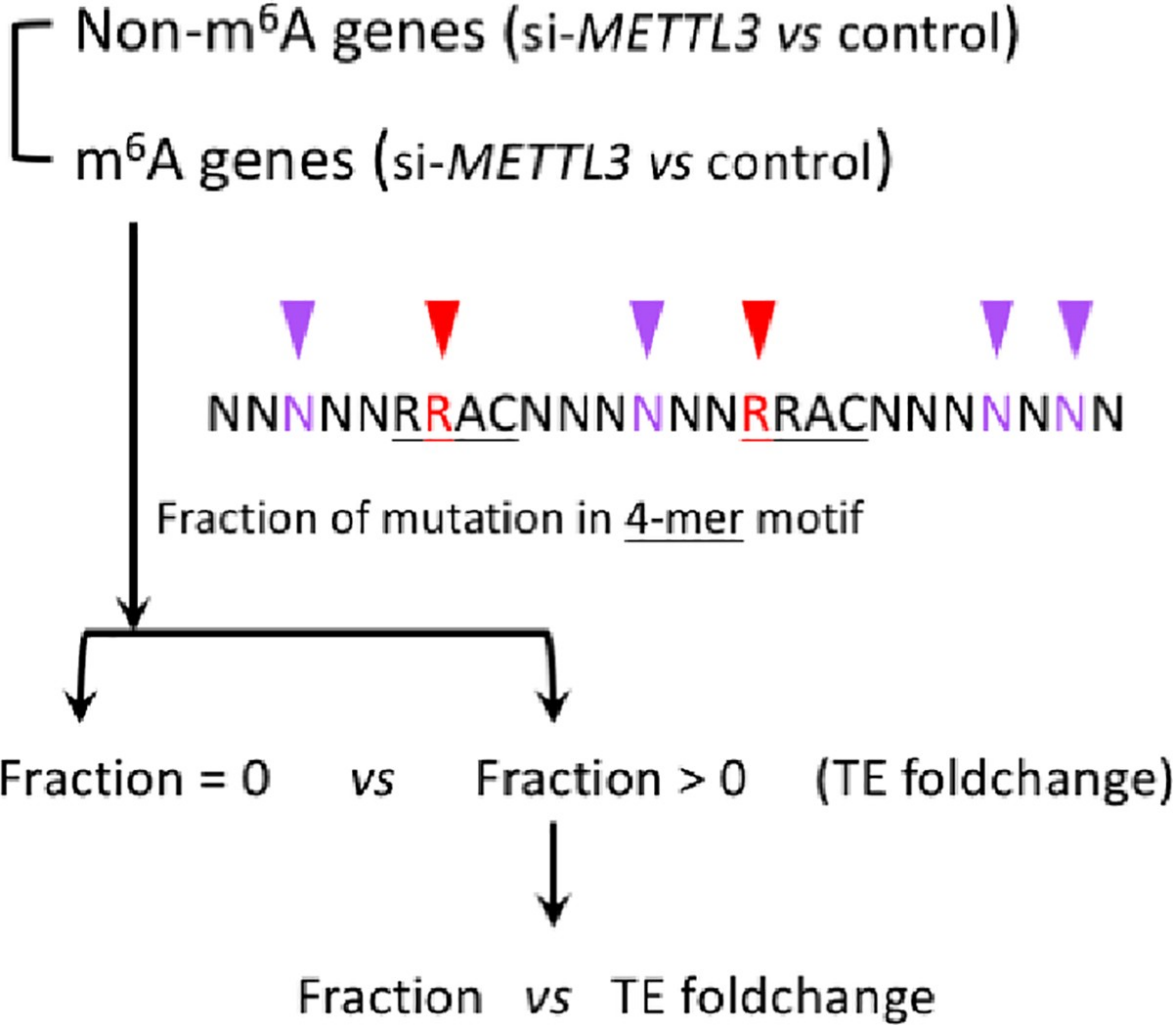
Foldchange of translation efficiency (TE) of m^6^A genes and non-m^6^A genes in si-*METTL3 versus* control. This is a workflow of the analysis in TE foldchange and fraction of mutations.

We profiled the TE foldchange of all genes in si-*METTL3* versus in the control samples (Fig 3A). If the TE of an m^6^A gene decreased when METTL3 was removed, then the gene was likely to be translationally up-regulated by m^6^A modification. Globally, the TE of the 6252 m^6^A genes was significantly down-regulated when METTL3 was removed (Fig 3B). Our finding was in agreement with the results of a previous study [6]. While the result here was expected by definition, it also served to validate our methodology involving the sequence alignment processes and the calculation of TE.

**Fig 4:**
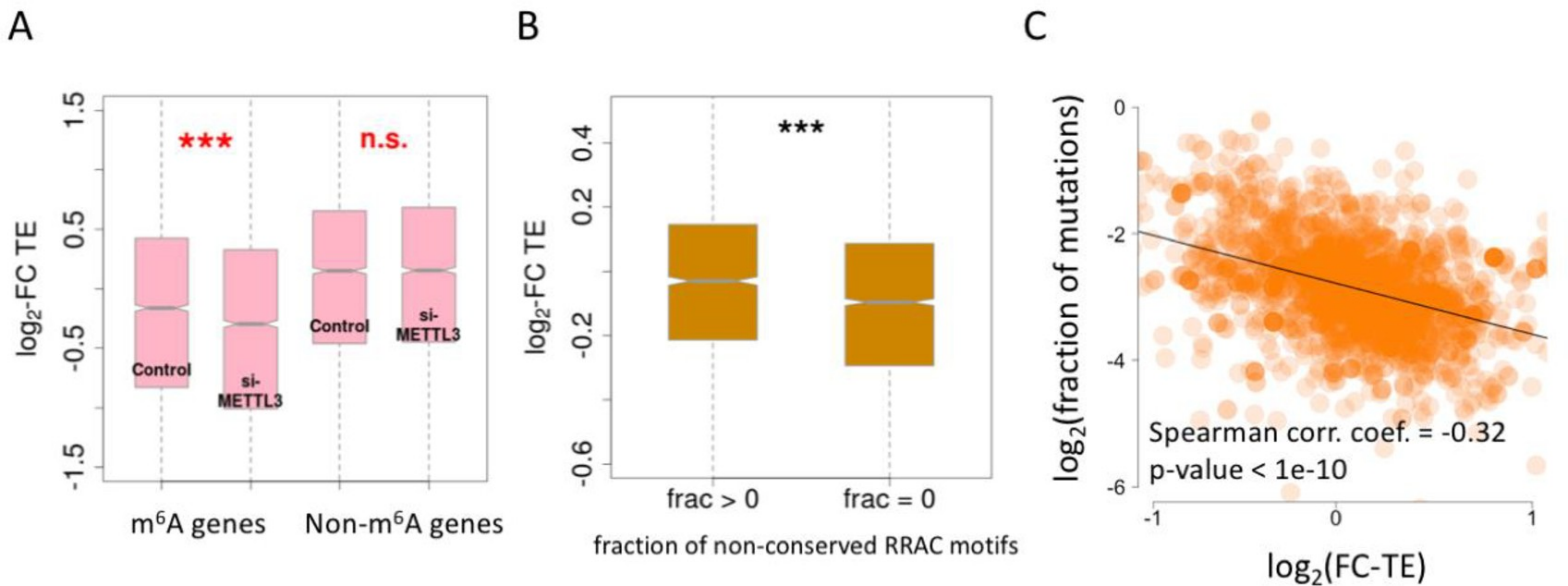
Correlation between TE foldchange (si-*METTL3 versus* control) and the fraction of mutations. (A) TE foldchange of m^6^A genes and non-m^6^A genes. (B) Boxplot comparing the TE foldchange of m^6^A genes with (fraction > 0) or without (fraction = 0) mutations in RRAC motifs. Also, fraction of mutations could be understood as fraction of non-conserved motifs. (C) Spearman’s correlation between TE foldchange (si-*METTL3 versus* control) and the fraction of mutations in RRAC motifs.

We next questioned whether the TE foldchange correlated with the fraction of mutations in the RRAC motifs (Fig 3A). Among the m^6^A genes, the genes without mutations in the RRAC motifs (fraction = 0) displayed greater changes in TE (lower log2 TE foldchange) compared to those with mutations in motifs (fraction > 0) (Fig 4A; each element in the boxplot represents a gene). Those genes with greater TE foldchange (si-*METTL3* versus control) were likely to have a closer connection with m^6^A regulation. In other words, if a gene relies more on m^6^A modification for the regulation of its TE, abolishing this crucial RRAC motif would be more harmful. Thus, our results indicated that genes that are highly regulated by m^6^A tend to have a lower fraction of mutations in the RRAC motifs. This inference was further supported by the negative correlation between the fraction of mutations in the RRAC motif and the TE foldchange (Fig 4B; each dot in the plot represents a gene). Although this result was expected, it also served to validate the reliability of our bioinformatic pipeline.

### Enrichment of A>G or G>A mutations in the RRAC motifs

As mentioned above, to conduct an unbiased comparison between the m^6^A modified and unmodified RRAC motifs, A>G and G>A mutations were excluded in all analyses. Since A>G and G>A mutations do not damage the RRAC (R = A or G) motif, if the m^6^A modified RRAC motifs are functionally more important than the unmodified RRAC motifs, then we would have observed higher proportions of A>G and G>A mutations in the m^6^A modified RRAC motifs.

**Fig 5:**
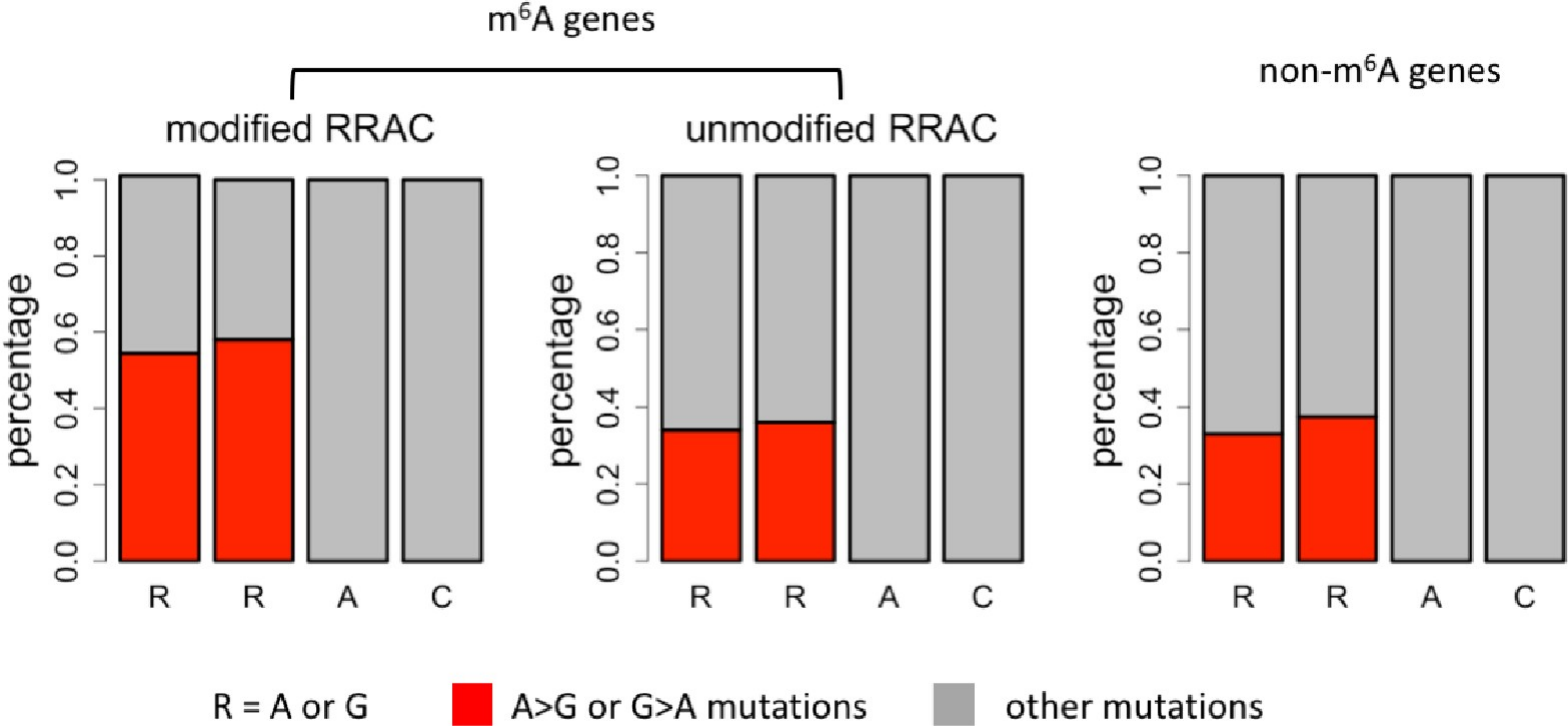
Percentage of A>G or G>A mutations in the first two positions (RR, R = A or G) of RRAC motifs.

We retrieved our previously excluded A>G or G>A mutations. For a particular site or region, we defined the percentage of A>G or G>A mutations as equal to the number of A>G or G>A mutations divided by number of all mutations. Three categories were classified for comparison: (1) m^6^A modified RRAC motifs in m^6^A genes, (2) unmodified RRAC motifs in m^6^A genes, and (3) RRAC motifs in non-m^6^A genes. The first two positions (RR, R = A or G) in this motif were investigated. The percentage of A>G or G>A mutations was remarkably higher in m^6^A modified RRAC motifs than in the other categories (Fig 5). This pattern supported our hypothesis that the A>G and G>A mutations are enriched in the first two positions of the RRAC motifs if the focal adenosine is methylated because these changes do not abolish the methylation propensity. This trend could be alternatively understood as the suppression of other mutation types in the RR positions that might cancel the possibility of methylation.

## Discussion

The mRNA translation regulation exerted by m^6^A modification is guaranteed by the proper methylation of adenosines in RRAC motifs. The disturbance of this motif would abolish the methylation process resulting in deleterious effects. Thus, compared to mutations in the other regions, mutations that abolish the RRAC motifs should be suppressed. In m^6^A genes of HeLa cells, there were fewer mutations in the RRAC motifs compared to mutations in the non-motif regions or the non-m^6^A genes. This trend was significant in the 5′UTR and 3′UTR regions. Since both 5′UTR and 3′UTR are able to regulate translation via m^6^A [6, 9, 21], we surmise that the difference in mutation fractions might be related to the m^6^A-mediated translational regulation. We first verified that compared to that of unmodified non-m^6^A genes, the translation of m^6^A modified genes was down-regulated in si-*METTL3* relative to that in the control [6]. We further revealed a negative correlation between the fraction of mutations in the RRAC motif and the TE foldchange of si-*METTL3* compared to that of control. This pattern indicated that genes strongly regulated by m^6^A have lower tolerance for mutations in the RRAC motifs.

We propose that the prevention of mutations in the RRAC motif might be a strategy adopted by cancer cells to maintain the elevated translation of m^6^A genes. Different cell/tissue types or cells/ tissues in the different developmental stages/conditions of organisms could have different m^6^A methylomes [3, 9]. Thus, maintenance of the methylome of a particular cell/tissue/stage is important for the integrity of cellular functions. If a novel mutation in HeLa cells (with low frequency in cell populations) abolishes the RRAC motif, the loss of the m^6^A modification as well as the loss of translation enhancement of the host gene would be slightly deleterious and will be eliminated by natural selection. However, mutations in other regions outside the RRAC motif would have a chance, albeit very slight, to be fixed by genetic drift. Thus, what we observed in the extant data was the reduced mutation fractions in the RRAC motifs in m^6^A genes.

For HeLa cells, the integrated methylome might guarantee the proper functioning of cellular processes. In other cancer cell types or tumor tissues/organs, their unique methylomes might even contribute to oncogenesis. The methylomes in different cancer/tumor types combined with the mutation data and translation data of the corresponding samples could clarify the selection force acting on the sequence features related to m^6^A. This knowledge would be helpful in determining the role of m^6^A modifications in translation control as well as their potential contribution to oncogenesis.

Regions outside the RRAC motifs of m^6^A genes and all regions in the non-m^6^A genes were treated as neutral regions that served as controls. However, many other functional sequence features, such as protein binding motifs, could be constrained and should exhibit avoidance of mutations. Our idea is to look only at one variable—RRAC motif—so that other variables have an equal chance to affect target regions like the RRAC motif or the control set located outside the motif. Thus, our observed patterns should be unbiased and robust.

## Conclusions

We propose that in HeLa cells, mutations in the RRAC motif would potentially stop the adenosines from being methylated and consequently prevent the enhancement of mRNA translation. These deleterious mutations in the RRAC motifs are suppressed in the 5′UTR and 3′UTR regions that are strongly associated with m^6^A-mediated translational regulation. This suppression is especially prominent in genes that are strongly regulated by m^6^A. This avoidance of mutations in the RRAC motif might be a strategy adopted by the HeLa cells to maintain the elevated translation of the m^6^A modified genes. The findings of this study increase the understanding of the dynamics of mutations affecting the m^6^A motifs and thus form the basis of further studies related to m^6^A.
